# Dynamic Changes and Prognostic Value of Gut Microbiota-Dependent Trimethylamine-N-Oxide in Acute Ischemic Stroke

**DOI:** 10.3389/fneur.2020.00029

**Published:** 2020-01-31

**Authors:** Chuhong Tan, Huidi Wang, Xuxuan Gao, Ruoting Xu, Xiuli Zeng, Ziming Cui, Jiajia Zhu, Qiheng Wu, Genghong Xia, Hongwei Zhou, Yan He, Jia Yin

**Affiliations:** ^1^Department of Neurology, Nanfang Hospital, Southern Medical University, Guangzhou, China; ^2^Department of General Surgery, Nanfang Hospital, Southern Medical University, Guangzhou, China; ^3^Division of Laboratory Medicine, Microbiome Medicine Center, Zhujiang Hospital, Southern Medical University, Guangzhou, China; ^4^State Key Laboratory of Organ Failure Research, Zhujiang Hospital, Southern Medical University, Guangzhou, China

**Keywords:** trimethylamine-N-oxide, acute ischemic stroke, dynamic changes, prognostic value, major ischemic events, unfavorable functional outcome

## Abstract

**Background:** Acute ischemic stroke (AIS) is an atherothrombotic disease. Trimethylamine-N-oxide (TMAO), a gut microbiota-dependent metabolite, has been shown to be proatherogenic and prothrombotic. However, the involvement of TMAO in AIS remains unclear. This study aimed to observe the dynamic changes of TMAO in AIS patients and identify the prognostic value of TMAO for major ischemic events and unfavorable functional outcomes.

**Methods:** This study included 204 AIS patients and 108 healthy controls. Blood samples for TMAO analyses were drawn at admission, 2 and 7 days of admission. Logistic regression models and receiver operating characteristic curves were established to identify associations between TMAO levels and major ischemic events (ischemic stroke, myocardial infarction, or death from an ischemic vascular event), as well as unfavorable functional outcomes (modified Rankin Scale score ≥3), at 90 days and 12 months.

**Results:** TMAO levels showed no significant changes before and within 24 h of AIS treatment (at admission) but decreased significantly thereafter. Elevated log_2_-transformed baseline TMAO levels were associated with increased risks of 90-day [odds ratio (OR), 2.62; 95% confidence interval (CI), 1.55–4.45; *p* < 0.001] and 12-month (OR, 3.59; 95% CI, 2.12–6.09; *p* < 0.001) major ischemic events, as well as 90-day (OR, 2.89; 95% CI, 1.46–5.71; *p* = 0.002) and 12-month (OR, 2.58; 95% CI, 1.50–4.46; *p* = 0.001) unfavorable functional outcomes, after adjustments for confounding factors. The areas under curve of baseline TMAO levels for predicting 90-day and 12-month major ischemic events were 0.72 (95% CI, 0.61–0.83; *p* < 0.001) and 0.76 (95% CI, 0.66–0.85; *p* < 0.001). Baseline TMAO levels improved the prognostic accuracy of conventional risk factors, National Institutes of Health Stroke Scale (NIHSS) score and N-terminal B-type natriuretic peptide (NT-proBNP) level.

**Conclusions:** TMAO levels decreased with time since stroke onset. Elevated TMAO levels at an earlier period portended poor stroke outcomes, broadening the potential clinical utility of TMAO as an independent prognostic marker and therapeutic target.

## Introduction

Despite guideline-based optimal treatments, acute ischemic stroke (AIS) levies a heavy burden, with high recurrence, and mortality rates (1). Early detection of a poor prognosis is pivotal for better care and allocation of medical resources. Pinpointing reported biomarkers with predictive power and clinical application remains challenging (2). Therefore, identifying potential markers associated independently with stroke outcome is urgently needed.

Trimethylamine-N-oxide (TMAO), a gut microbiota-dependent metabolite derived from dietary nutrients (phosphatidylcholine, choline and L-carnitine), has been reported to promote atherosclerosis (3), foster platelet hyperreactivity and enhance thrombotic potential (4). Meanwhile, a higher TMAO level was found to correlate with an increased risk of cardiovascular events independent of traditional risk factors (5–17). However, most prior studies were from Western countries; studies in Asian populations, whose dietary patterns differ from those of Westerners, are limited. In addition, most published studies were conducted on patients with cardiovascular diseases. The dynamic changes and potential associations of TMAO in AIS, another atherothrombotic disease, are unclear.

In our previous study, the fasting plasma TMAO levels in AIS patients within 7 days of onset were lower than those in healthy controls (18), which we speculated that either the stroke event or the AIS treatment might reduce TMAO. Another study included AIS patients within 24 h of symptom onset and found higher TMAO levels (19). Low-dose aspirin, a widely used antithrombotic agent for AIS, could attenuate the elevated TMAO (20). So far, little was known about the dynamic changes of TMAO after AIS, which might be associated with prognosis. Therefore, we sought to observe the dynamic changes in TMAO levels before and after AIS treatment and identify the prognostic value of TMAO for major ischemic events (ischemic stroke, myocardial infarction or death from an ischemic vascular event) and unfavorable functional outcomes (modified Rankin Scale Score ≥3) at 90 days and 12 months in this prospective observational cohort.

## Materials and Methods

### Participants and Study Design

**Figure 1:**
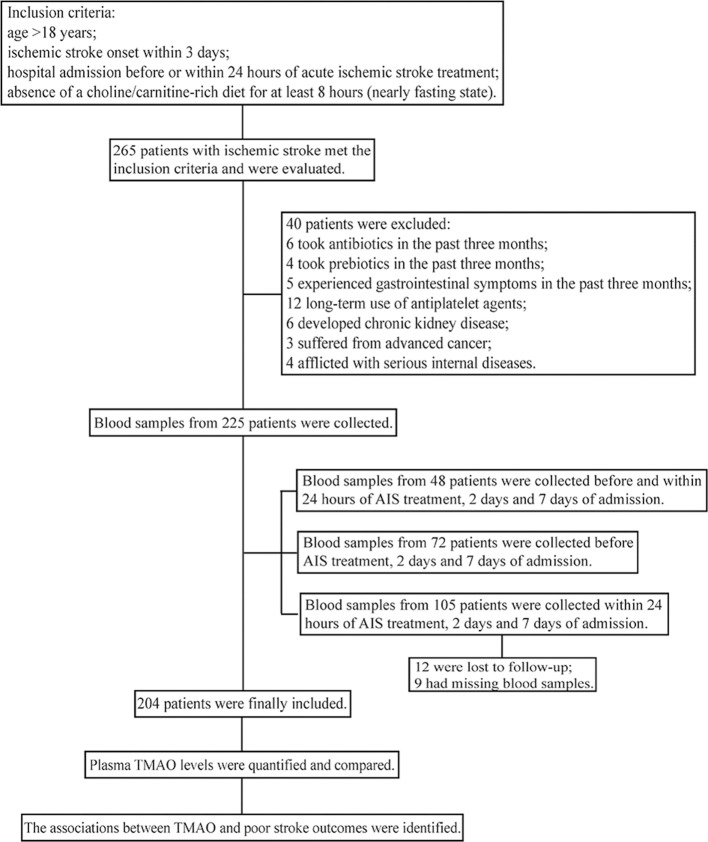
Flow chart of the study.

This study was conducted at the Department of Neurology in Nanfang Hospital of Southern Medical University (Guangzhou, China) from May 2017 to February 2018. A flow chart of the study is shown in Figure 1. The inclusion criteria of our study were as follows: (1) AIS patients aged >18 years; (2) within 3 days of onset; (3) had not yet received any AIS treatment (neither revascularization therapies nor antiplatelet agents) or had already received treatments but within 24 h; (4) no choline/carnitine-rich diet for at least 8 h (nearly fasting state). Patients who developed chronic kidney disease/advanced cancer/serious internal diseases, had a history of taking antiplatelet agents/antibiotics/prebiotics or experienced gastrointestinal symptoms in the past 3 months were excluded. Healthy controls aged >18 years; without a history of stroke, atrial fibrillation, coronary heart disease or chronic kidney disease; and had not used antibiotics/prebiotics or experienced gastrointestinal symptoms in the past 3 months, were recruited from the Bureau of Agricultural Reclamation (Guangzhou, China). All participants provided written informed consent in accordance with the Declaration of Helsinki. This study was approved by the investigational review board of Nanfang Hospital, Southern Medical University (NFEC-2016-148) and was registered at http://www.chictr.org (registration number: ChiCTR-ROC-17011567).

The diagnosis and treatment of acute ischemic stroke were in accordance with the Chinese guidelines (21). Demographic characteristics, history of risk factors and clinical features were collected at enrollment. The National Institutes of Health Stroke Scale (NIHSS) score was used to evaluate stroke severity, and the modified Rankin Scale (mRS) score was used to evaluate functional outcome. Ischemic stroke was classified as large artery atherosclerosis (thrombotic), small artery occlusion lacunae (lacunar), cardiac embolism (embolic), other course, and unknown, according to the TOAST classification system. Data were acquired and assessed by experienced neurologists. All patients underwent AIS treatment with revascularization therapies (intravenous thrombolysis and/or endovascular treatments), antiplatelet agents (aspirin/clopidogrel/aspirin + clopidogrel), statins, hypotensive or hypoglycemic drugs if necessary, according to the latest guidelines (21) since the time of entry; and were requested to continue the guideline-recommended secondary prevention therapies post-discharge. The primary end point was major ischemic event within 90 days and 12 months, defined as ischemic stroke, myocardial infarction, or death from an ischemic vascular event. The secondary end point was unfavorable functional outcome (mRS score ≥3) within 90 days and 12 months. End points were evaluated by 2 trained staff members blinded to the TMAO concentrations via structured follow-up telephone interviews.

### Blood Collection and Laboratory Testing

The first blood samples of AIS patients were collected at admission [when patients had not yet received any AIS treatment (neither revascularization therapies nor antiplatelet agents) or had already received treatments but within 24 h]. First, to examine the dynamic changes of TMAO levels during the early period after admission, blood samples were collected successively (before AIS treatment, within 24 h of treatment, 2 and 7 days of admission) in a pilot study. Then we enrolled a larger cohort, in which blood samples were collected at admission (before or within 24 h of AIS treatment), 2 and 7 days of admission, to investigate the dynamic changes of TMAO. Blood samples were drawn once from healthy controls at 6:30 am (fasting state), those from AIS patients were drawn at admission (no choline/carnitine-rich diet for at least 8 h), 6:30 am within 24 h of treatment (those included in the pilot study, fasting state), 6:30 am at days 2 and 7 (fasting state). Routine laboratory tests were performed for all enrolled participants via standard detection methods. The estimated glomerular filtration rate (eGFR) was calculated by a formula specific to the Chinese population: eGFR (ml/min/1.73 m^2^) = 186 × creatinine^−1.154^ × age^−0.203^ × 1.233 × (0.742, female) (22).

### Quantification of Plasma TMAO Levels

The collected blood samples were immediately processed and stored at −80°C until analysis. Plasma TMAO levels were quantified by stable isotope dilution liquid chromatography tandem mass spectrometry (6460 Series Triple Quadrupole LC/MS; Agilent) using a d9-(trimethyl)-labeled internal standard, as previously described (23).

In total, 50 μL of plasma was added to a 1.5 mL Eppendorf tube and mixed with 200 μL of 10 μmol/L internal standard composed of d9-TMAO in methanol. The samples were vortexed for 1 min, and then the supernatant was aliquoted following centrifugation at 15,000 g and 4°C for 25 min. Two microliters of supernatant was injected directly into a silica column (4.69 × 250 mm, 5 μm Luna silica, catalog no. 00G-4274-E0; Phenomenex) at a flow rate of 0.5 mL/min^−1^ with 80% solvent A (0.1% formic acid in water) and 20% solvent B (methanol). TMAO and d9-TMAO were monitored in the positive multiple reaction monitoring mass spectrometry mode using characteristic precursor–production transitions including *m/z* 76/58 and *m/z* 85/66, respectively. To calculate TMAO concentrations, serial concentrations of TMAO standard were added to control plasma to prepare a calibration curve.

### Statistical Analysis

The results are expressed as number (percentage, %) for categorical variables and as median (interquartile range, IQR) for continuous variables. Categorical variables were compared by the χ^2^ test or the Fisher exact test, and continuous variables by the Mann-Whitney *U*-test or the Kruskal-Wallis *H*-test. The TMAO levels showed a skewed distribution, so the Wilcoxon-rank sum test was used for the comparison of paired TMAO samples at different time points. To investigate the associations between TMAO (log_2_-transformed) and major ischemic events, as well as unfavorable functional outcomes, both univariate and multivariate logistic regression models were established. The multivariate models included all variables with statistical significance (*p* < 0.10) in the univariate model, as well as those with clinical significance. Receiver operating characteristic (ROC) curves were used to examine the prognostic ability of TMAO, and the results are reported as areas under the curves (AUCs). The comparison of AUCs was conducted with a method proposed by Delong et al. (24) using MedCalc statistical software version 18.11.3. Other analyses were performed with IBM SPSS statistical software version 22.0 (SPSS Inc., Chicago, IL, USA), and a 2-tailed *p* < 0.05 was considered statistically significant.

## Results

### Baseline Characteristics of the Study Participants

According to the inclusion and exclusion criteria, 204 eligible AIS patients and 108 healthy controls were finally included in this study. Details of the enrollment process are shown in Figure 1 and Supplementary Figure 1. The median age of the AIS patients was 59 years, and 66.7% were male. The median NIHSS score was 4. According to the symptoms and imaging data, 96 AIS patients were thrombotic, 49 were lacunar, 32 were embolic, 7 were classified as other and 20 were classified as unknown. Demographic and clinical parameters of cases and controls are shown in Supplementary Table 1. The two groups had no significant differences in age, sex or history of smoking.

Primary outcomes at 90 days after discharge included recurrent ischemic stroke in 10 patients (4.9%), death from an ischemic vascular event in 16 patients (7.8%) and a composite of major ischemic events in 26 patients (12.7%); primary outcomes at 12 months after discharge included recurrent ischemic stroke in 16 patients (7.8%), death from an ischemic vascular event in 21 patients (10.3%) and a composite of major ischemic events in 37 patients (18.1%). Patients who developed major ischemic events were older (median, 64.5 vs. 59, *p* = 0.026 for 90 days and 66 vs. 59, *p* = 0.013 for 12 months); were subjected more to intravenous thrombolysis + endovascular treatments (38.5 vs. 15.2%, *p* = 0.004 for 90 days and 35.1 vs. 14.4%, *p* = 0.003 for 12 months); exhibited more severe stroke (median NIHSS, 10 vs. 4, *p* = 0.010 for 90 days and 8 vs. 4, *p* = 0.022 for 12 months), more cardioembolism (30.8 vs. 13.5%, *p* = 0.024 for 90 days and 27.0 vs. 13.2%, *p* = 0.036 for 12 months) and more dysphagia (61.5 vs. 28.7%, *p* = 0.001 for 90 days and 51.4 vs. 28.7%, *p* = 0.008 for 12 months); had greater prevalences of atrial fibrillation (34.6 vs. 10.1%, *p* = 0.001 for 90 days and 29.7 vs. 9.6%, *p* = 0.001 for 12 months) and prior stroke (30.8 vs. 12.4%, *p* = 0.013 for 90 days and 24.3 vs. 12.6%, *p* = 0.068 for 12 months); and had higher levels of neutrophils (NEU, median, 6.56 vs. 5.49 × 10^9^/L, *p* = 0.020 for 90 days and 6.36 vs. 5.46 × 10^9^/L, *p* = 0.013 for 12 months), N-terminal B-type natriuretic peptide (NT-proBNP, median, 294.40 vs. 77.47 pg/ml, *p* = 0.001 for 90 days and 181.70 vs. 71.73 pg/ml, *p* = 0.003 for 12 months) and total cholesterol (TC, median, 5.31 vs. 4.75 mmol/L, *p* = 0.027 for 90 days and 5.17 vs. 4.75 mmol/L, *p* = 0.18 for 12 months) than those who did not suffered from major ischemic events (Tables 1, 2).

**Table 1:** Baseline characteristics of the AIS patients with or without major ischemic events at 90 days.

**Characteristics**	**AIS patients**	**Major ischemic events at 90 days**		***p*-value**
		**Yes**	**No**	
No. of subjects	204	26	178	
**Demographics**				
Male	136 (66.7)	16 (61.5)	120 (67.4)	0.55
Age, y	59 (21)	64.5 (20)	59 (19)	0.026
History of smoking	79 (38.7)	10 (38.5)	69 (38.8)	0.98
**Medical history**				
History of hypertension	134 (65.7)	16 (61.5)	118 (66.3)	0.63
History of diabetes	59 (28.9)	7 (26.9)	52 (29.2)	0.81
History of atrial fibrillation	27 (13.2)	9 (34.6)	18 (10.1)	0.001
History of coronary heart disease	18 (8.8)	4 (15.4)	14 (7.9)	0.21
History of stroke	30 (14.7)	8 (30.8)	22 (12.4)	0.013
History of dyslipidemia	76 (37.3)	12 (46.2)	64 (36.0)	0.13
**Clinical features**				
NIHSS score	4 (9)	10 (19)	4 (7)	0.010
Dysphagia	67 (32.8)	16 (61.5)	51 (28.7)	0.001
**Revascularization therapies**				0.037
Conventional treatments	140 (68.6)	14 (53.8)	126 (70.8)	
Intravenous thrombolysis	16 (7.8)	1 (3.8)	15 (8.4)	
Intravenous thrombolysis + endovascular treatments	37 (18.1)	10 (38.5)	27 (15.2)	
Endovascular treatments	11 (5.4)	1 (3.8)	10 (5.6)	
**Stroke causes**				0.034
Large artery atherosclerosis	96 (47.1)	10 (38.5)	86 (48.3)	
Small artery occlusion lacunae	49 (24.0)	3 (11.5)	46 (25.8)	
Cardiac embolism	32 (15.7)	8 (30.8)	24 (13.5)	
Other cause	7 (3.4)	0	7 (3.9)	
Unknown	20 (9.8)	5 (19.2)	15 (8.4)	
**Antiplatelet agents**				0.65
Aspirin	46 (22.6)	6 (23.1)	40 (22.5)	
Clopidogrel	49 (24.0)	8 (30.8)	41 (23.0)	
Aspirin + clopidogrel	109 (53.4)	12 (46.2)	97 (54.5)	
**Laboratory findings**				
NEU, × 10^9^/L	5.64 (3.90)	6.56 (4.94)	5.49 (3.76)	0.020
NT-proBNP, pg/ml	88.07 (376.49)	294.40 (1620.47)	77.47 (272.52)	0.001
TC, mmol/L	4.89 (1.45)	5.31 (0.86)	4.75 (1.50)	0.027
eGFR, ml/min/1.73 m^2^	100.95 (40.64)	96.62 (31.17)	101.85 (40.37)	0.32

*Data are presented as number (%) or median (IQR)*.

**Table 2:** Baseline characteristics of the AIS patients with or without major ischemic events at 12 months.

**Characteristics**	**AIS patients**	**Major ischemic events at 12 months**		***p*-value**
		**Yes**	**No**	
No. of subjects	204	37	167	
**Demographics**				
Male	136 (66.7)	23 (62.2)	113 (67.7)	0.52
Age, y	59 (21)	66 (23)	59 (18)	0.013
History of smoking	79 (38.7)	11 (29.7)	68 (40.7)	0.21
**Medical history**				
History of hypertension	134 (65.7)	24 (64.9)	110 (65.9)	0.91
History of diabetes	59 (28.9)	11 (29.7)	48 (28.7)	0.91
History of atrial fibrillation	27 (13.2)	11 (29.7)	16 (9.6)	0.001
History of coronary heart disease	18 (8.8)	5 (13.5)	13 (7.8)	0.27
History of stroke	30 (14.7)	9 (24.3)	21 (12.6)	0.068
History of dyslipidemia	76 (37.3)	15 (40.5)	61 (36.5)	0.65
**Clinical features**				
NIHSS score	4 (9)	8 (16)	4 (7)	0.022
Dysphagia	67 (32.8)	19 (51.4)	48 (28.7)	0.008
**Revascularization therapies**				0.020
Conventional treatments	140 (68.6)	22 (59.5)	118 (70.7)	
Intravenous thrombolysis	16 (7.8)	1 (2.7)	15 (9.0)	
Intravenous thrombolysis + endovascular treatments	37 (18.1)	13 (35.1)	24 (14.4)	
Endovascular treatments	11 (5.4)	1 (2.7)	10 (6.0)	
**Stroke causes**				0.086
Large artery atherosclerosis	96 (47.1)	15 (40.6)	81 (48.5)	
Small artery occlusion lacunae	49 (24.0)	5 (13.5)	44 (26.3)	
Cardiac embolism	32 (15.7)	10 (27.0)	22 (13.2)	
Other cause	7 (3.4)	1 (2.7)	6 (3.6)	
Unknown	20 (9.8)	6 (16.2)	14 (8.4)	
**Antiplatelet agents**				0.033
Aspirin	46 (22.6)	6 (16.2)	40 (23.9)	
Clopidogrel	49 (24.0)	15 (40.6)	34 (20.4)	
Aspirin + clopidogrel	109 (53.4)	16 (43.2)	93 (55.7)	
**Laboratory findings**				
NEU, × 10^9^/L	5.64 (3.90)	6.36 (4.41)	5.46 (3.77)	0.013
NT-proBNP, pg/ml	88.07 (376.49)	181.70 (1467.65)	71.73 (275.19)	0.003
TC, mmol/L	4.89 (1.45)	5.17 (1.28)	4.75 (1.49)	0.18
eGFR, ml/min/1.73 m^2^	100.95 (40.64)	98.61 (35.19)	101.60 (40.07)	0.55

*Data are presented as number (%) or median (IQR)*.

After 90 days and 12 months of follow-up, a total of 54 (26.5%) and 38 (18.6%) patients experienced unfavorable functional outcomes (mRS score ≥3). Patients who experienced unfavorable functional outcomes were older (median, 66.5 vs. 57.5, *p* < 0.001 for 90 days and 67 vs. 59, *p* = 0.001 for 12 months); were subjected more to intravenous thrombolysis + endovascular treatments (53.7 vs. 5.3%, *p* < 0.001 for 90 days and 55.3 vs. 9.6%, *p* < 0.001 for 12 months); exhibited more severe stroke (median NIHSS, 14 vs. 3, *p* < 0.001 for 90 days and 15 vs. 3, *p* < 0.001 for 12 months), more cardioembolism (33.3 vs. 9.3%, *p* < 0.001 for 90 days and 36.8 vs. 10.8%, *p* < 0.001 for 12 months) and more dysphagia (87.0 vs. 13.3%, *p* < 0.001 for 90 days and 92.1 vs. 19.3%, *p* < 0.001 for 12 months); had greater prevalences of atrial fibrillation (38.9 vs. 4.0%, *p* < 0.001 for 90 days and 44.7 vs. 6.0%, *p* < 0.001 for 12 months) and coronary heart disease (18.5 vs. 5.3%, *p* = 0.003 for 90 days and 18.4 vs. 6.6%, *p* = 0.021 for 12 months); and had higher levels of NEU (median, 6.69 vs. 5.37 × 10^9^/L, *p* = 0.001 for 90 days and 6.69 vs. 5.45 × 10^9^/L, *p* = 0.004 for 12 months) and NT-proBNP (median, 233.65 vs. 68.28 pg/ml, *p* < 0.001 for 90 days and 279.45 vs. 71.73 pg/ml, *p* = 0.001 for 12 months) than those who did not suffered from unfavorable functional outcomes (Supplementary Tables 2, 3).

### Dynamic Changes of TMAO Levels in AIS Patients During Treatment

**Figure 2:**
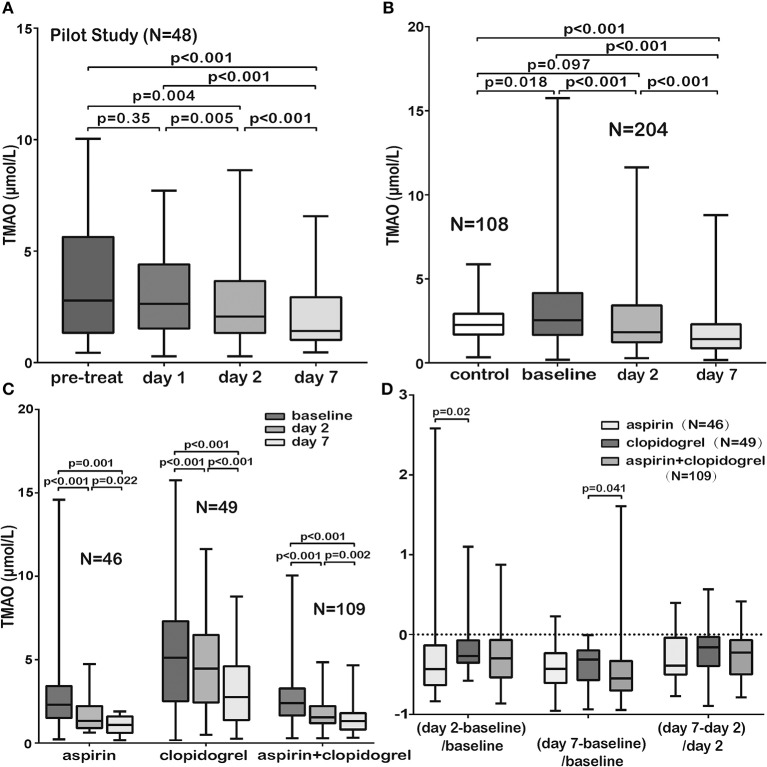
Comparison of TMAO levels in different subgroups. **(A)** The TMAO levels in AIS patients (pilot study, *N* = 48) showed no significant changes before and within 24 h of AIS treatment but decreased significantly 24 h later. **(B)** The baseline TMAO levels in AIS patients were higher than those in controls. The TMAO levels decreased significantly, with levels lower than those of the controls at day 7. **(C)** TMAO levels in the three groups administered guideline-based antiplatelet treatment (aspirin/clopidogrel/aspirin + clopidogrel) decreased significantly. **(D)** The patients administered aspirin or aspirin + clopidogrel showed a sharper drop in TMAO (measurement: latter TMAO levels minus earlier levels and then divided by earlier levels). Pre-treat indicated before AIS treatment; day 1, within 24 h of treatment; day 2, 2 days of admission and day 7, 7 days of admission.

Firstly, blood samples from 48 patients were collected successively (before AIS treatment, within 24 h of treatment, 2 and 7 days of admission) to examine the dynamic changes of TMAO levels during the early period after admission. The results indicated that the TMAO levels showed no significant changes before and within 24 h of treatment but decreased significantly 24 h later (Figure 2A). We then defined the TMAO levels before or within 24 h of AIS treatment as the baseline levels and explored the levels in a larger cohort to investigate the dynamic changes of TMAO (at admission, 2 and 7 days of admission). Finally, blood samples from 72 patients were collected before treatment, 2 and 7 days of admission; those from 105 patients were collected within 24 h of treatment, 2 and 7 days of admission, of which 12 were lost to follow-up and 9 had missing blood samples. In total, 204 patients were included. We found that the baseline TMAO levels were higher in AIS patients than in controls (median, 2.54 vs. 2.26 μmol/L, *p* = 0.018, Figure 2B) and that TMAO levels decreased significantly after stroke, with levels lower than those of the controls at day 7 (Figure 2B).

No significant differences were observed in baseline TMAO levels among patients with different TOAST subtypes (*p* = 0.39), and no significant correlation was observed between baseline TMAO levels and NIHSS scores (*p* = 0.38). Interestingly, different dietary habits of neither AIS patients nor healthy controls showed significant difference on TMAO levels (Supplementary Figures 2, 3, *p* = 0.95 for AIS patients and *p* = 0.29 for healthy controls). No significant differences were found on TMAO levels among AIS patients receiving different revascularization therapies (Supplementary Figure 4, conventional/intravenous thrombolysis/endovascular treatments/both intravenous thrombolysis and endovascular treatments, *p* = 0.27) but patients subjected to both intravenous thrombolysis and endovascular treatments tended to suffer more major ischemic events and unfavorable functional outcomes than those subjected to other therapies (*p* = 0.004 for 90 days, *p* = 0.003 for 12 months and *p* < 0.001 for 90 days, *p* < 0.001 for 12 months). AIS is an atherothrombotic disease. Antithrombotic agents are one of the most important part for stroke prevention. It remains uncertain the impact of different antithrombotic agents on TMAO levels. Our results showed that TMAO levels in the three groups administered guideline-based antiplatelet treatment [aspirin (*N* = 46)/clopidogrel (*N* = 49)/aspirin + clopidogrel (*N* = 109)] decreased significantly (Figure 2C). The patients who received aspirin or aspirin + clopidogrel showed a sharper drop in TMAO levels (Figure 2D). The patients who received clopidogrel suffered more 12-month major ischemic events (*p* = 0.033), although no difference was found in 90-day major ischemic events (*p* = 0.65). However, the patients who received aspirin experienced more unfavorable functional outcomes at 90 days (*p* < 0.001) and at 12 months (*p* = 0.003).

### Associations Between TMAO and Poor Stroke Outcomes

**Figure 3:**
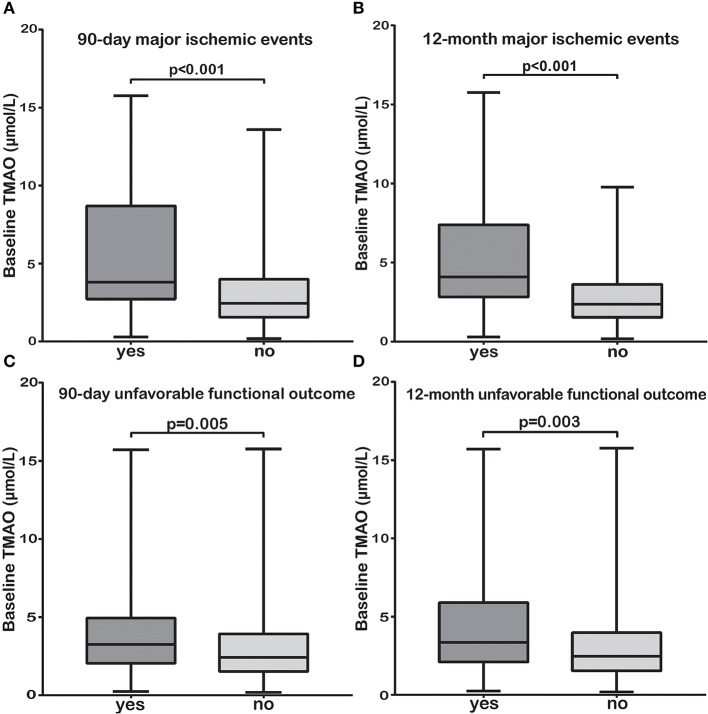
The TMAO levels before or within 24 h of AIS treatment (baseline) were significantly higher in patients with 90-day or 12-month major ischemic events **(A,B)** and unfavorable functional outcomes **(C,D)** than in those without.

We sought to explore the associations between baseline TMAO levels (with little influence by stroke course and treatment) and poor stroke outcomes. Subjects with 90-day or 12-month major ischemic events and unfavorable functional outcomes had significantly higher baseline TMAO levels than those without (median, 3.80 vs. 2.45 μmol/L, *p* < 0.001; median, 4.09 vs. 2.37 μmol/L, *p* < 0.001 and median, 3.25 vs. 2.42 μmol/L, *p* = 0.005; median, 3.35 vs. 2.47 μmol/L, *p* = 0.003, respectively, Figures 3A–D). The relationships between baseline TMAO levels and major ischemic events, as well as unfavorable functional outcomes, are shown in Table 3 and Supplementary Tables 4, 5. Log_2_-transformed baseline TMAO levels had ORs of 2.21 (Model 1, 95% CI, 1.41–3.47; *p* = 0.001) for 90-day major ischemic events, 2.74 (Model 1, 95% CI, 1.78–4.21; *p* < 0.001) for 12-month major ischemic events, 1.44 (Model 1, 95% CI, 1.06–1.97; *p* = 0.02) for 90-day unfavorable functional outcomes and 1.58 (Model 1, 95% CI, 1.10–2.26; *p* = 0.012) for 12-month unfavorable functional outcomes.

**Table 3:** Odds ratios and 95% confidence intervals of clinical outcomes according to log_2_-transformed baseline TMAO levels in acute ischemic stroke.

	**No. of cases (%)**	**Model 1**		**Model 2**		**Model 3**		**Model 4**	
		**OR** **(95% CI)**	***p*-value**	**OR** **(95% CI)**	***p*-value**	**OR** **(95% CI)**	***p*-value**	**OR** **(95% CI)**	***p*-value**
**Major ischemic events at 90 days**									
Baseline TMAO (log_2_-transformed)	26 (12.7)	2.21 (1.41–3.47)	0.001	2.26 (1.41–3.60)	0.001	2.32 (1.43–3.78)	0.001	2.62 (1.55–4.45)	<0.001
**Major ischemic events at 12 months**									
Baseline TMAO (log_2_-transformed)	37 (18.1)	2.74 (1.78–4.21)	<0.001	2.80 (1.80–4.36)	<0.001	3.04 (1.91–4.84)	<0.001	3.59 (2.12–6.09)	<0.001
**Unfavorable functional outcome (mRS score** **≥3) at 90 days**									
Baseline TMAO (log_2_-transformed)	54 (26.5)	1.44 (1.06–1.97)	0.02	1.43 (1.02–2.01)	0.039	2.33 (1.31–4.14)	0.004	2.89 (1.46–5.71)	0.002
**Unfavorable functional outcome (mRS score** **≥3) at 12 months**									
Baseline TMAO (log_2_-transformed)	38 (18.6)	1.58 (1.10–2.26)	0.012	1.67 (1.14–2.44)	0.009	2.18 (1.30–3.68)	0.003	2.58 (1.50–4.46)	0.001

*Model 1: unadjusted*.

*Model 2: adjusted for age; sex; history of smoking, hypertension, diabetes, atrial fibrillation, coronary heart disease, stroke and dyslipidemia; and eGFR*.

*Model 3: adjusted for all factors in model 2, plus revascularization therapies, stroke etiologies, dysphagia, NIHSS score and antiplatelet agents*.

*Model 4: adjusted for all factors in model 3, plus NEU, NT-proBNP and TC levels*.

Patients with higher log_2_-transformed baseline TMAO levels had an increased risk of 90-day (Model 2, OR, 2.26; 95% CI, 1.41–3.60; *p* = 0.001), 12-month (Model 2, OR, 2.80; 95% CI, 1.80–4.36; *p* < 0.001) major ischemic events and 90-day (Model 2, OR, 1.43; 95% CI, 1.02–2.01; *p* = 0.039), 12-month (Model 2, OR, 1.67; 95% CI, 1.14–2.44; *p* = 0.009) unfavorable functional outcomes after adjustments for age; sex; history of smoking, hypertension, diabetes, atrial fibrillation, coronary heart disease, stroke, and dyslipidemia; and eGFR. This increase persisted even after further adjustments for revascularization therapies, stroke etiologies, dysphagia, NIHSS score, antiplatelet agents (Model 3, OR, 2.32; 95% CI, 1.43–3.78; *p* = 0.001 for 90-day; OR, 3.04; 95% CI, 1.91–4.84; *p* < 0.001 for 12-month major ischemic events and OR, 2.33; 95% CI, 1.25–4.33; *p* = 0.007 for 90-day; OR, 2.18; 95% CI, 1.30–3.68; *p* = 0.003 for 12-month unfavorable functional outcomes), and for NEU, NT-proBNP, TC levels (Model 4, OR, 2.62; 95% CI, 1.55–4.45; *p* < 0.001 for 90-day; OR, 3.59; 95% CI, 2.12–6.09; *p* < 0.001 for 12-month major ischemic events and OR, 2.89; 95% CI, 1.46–5.71; *p* = 0.002 for 90-day; OR, 2.58; 95% CI, 1.50–4.46; *p* = 0.001 for 12-month unfavorable functional outcomes). Neither the log_2_-transformed TMAO levels at day 2 nor those at day 7 were associated with 90-day major ischemic events, 90-day unfavorable functional outcomes and 12-month unfavorable functional outcomes (*p* = 0.28, *p* = 0.38; *p* = 0.61, *p* = 0.83 and *p* = 0.053, *p* = 0.062, respectively), while both were correlated with 12-month major ischemic events (OR, 2.09; 95% CI, 1.32–3.29; *p* = 0.002 and OR, 2.14; 95% CI, 1.20–3.81; *p* = 0.01, respectively).

### Prognostic Value of TMAO for Poor Stroke Outcomes

**Figure 4:**
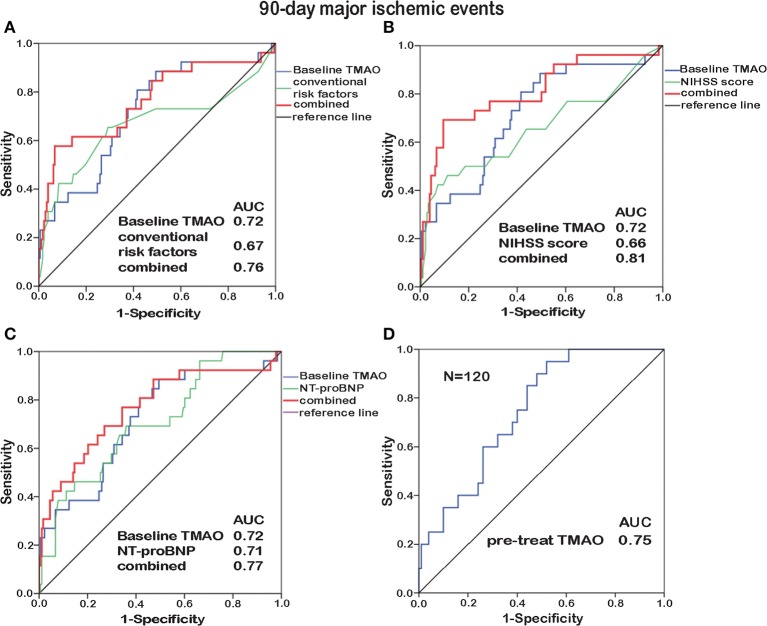
ROC curve analyses relating baseline TMAO levels and conventional risk factors (hypertension, diabetes, atrial fibrillation, coronary heart disease, dyslipidemia, and prior stroke; **A**), the NIHSS scores **(B)**, the NT-proBNP levels **(C)**, as well as the TMAO levels before treatment (pre-treat, *N* = 120, **D**), to 90-day major ischemic events. Elevated baseline TMAO levels played a moderate predictive role (AUC = 0.72) in 90-day major ischemic events. Combining baseline TMAO levels with the NIHSS scores resulted in higher AUCs (*p* = 0.016). The TMAO levels before treatment yielded additive prognostic value (AUC = 0.75).

**Figure 5:**
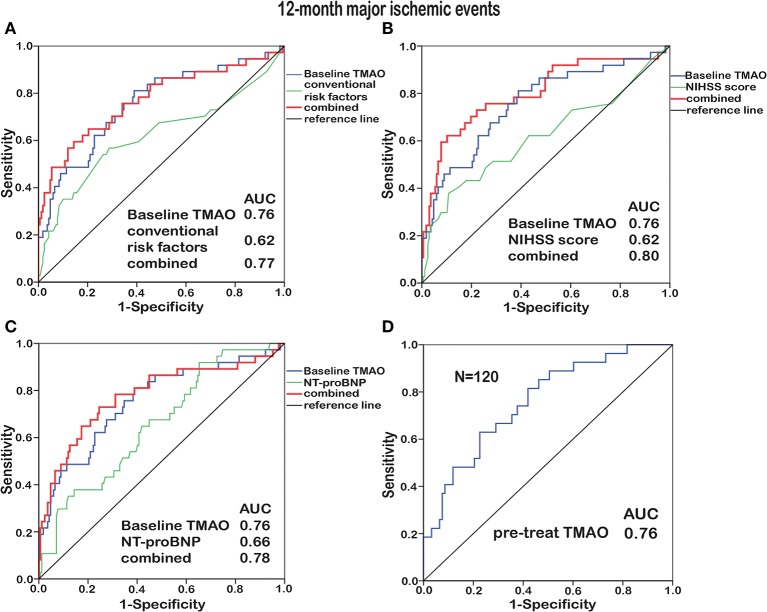
ROC curve analyses relating baseline TMAO levels and conventional risk factors (hypertension, diabetes, atrial fibrillation, coronary heart disease, dyslipidemia, and prior stroke; **A**), the NIHSS scores **(B)**, the NT-proBNP levels **(C)**, as well as the TMAO levels before treatment (pre-treat, *N* = 120, **D**), to 12-month major ischemic events. Elevated baseline TMAO levels played a moderate predictive role (AUC = 0.76) in 12-month major ischemic events. Combining baseline TMAO levels with conventional risk factors/the NIHSS scores/the NT-proBNP levels resulted in higher AUCs (*p* = 0.016, *p* < 0.001, and *p* = 0.012, respectively).

To identify the prognostic potential of baseline TMAO for major ischemic events, ROC curves were analyzed (Figures 4A–C, 5A–C). With AUCs of 0.72 (95% CI, 0.61–0.83; *p* < 0.001) and 0.76 (95% CI, 0.66–0.85; *p* < 0.001), baseline TMAO levels tended to show better discriminatory ability for 90-day and 12-month major ischemic events compared with conventional risk factors, including hypertension, diabetes, atrial fibrillation, coronary heart disease, dyslipidemia, and prior stroke; the NIHSS scores and the NT-proBNP levels. Moreover, baseline TMAO levels improved the prognostic accuracy of the NIHSS scores for 90-day major ischemic events (*p* = 0.016) and that of conventional risk factors, the NIHSS scores and the NT-proBNP levels for 12-month major ischemic events (*p* = 0.016, *p* < 0.001, and *p* = 0.012, respectively). Nevertheless, baseline TMAO levels did not have markedly superior predictive values for 90-day or 12-month unfavorable functional outcomes, compared with conventional risk factors, the NIHSS scores and the NT-proBNP levels, with AUCs of 0.65 (95% CI, 0.54–0.71; *p* = 0.005) and 0.65 (95% CI, 0.56–0.75; *p* = 0.003), respectively (Supplementary Table 6).

Subjects enrolled before stroke treatment (*N* = 48 + 72) were selected for inclusion in another ROC curve analysis, which revealed that the TMAO levels before treatment exhibited additive prognostic value for major ischemic events (Figures 4D, 5D). No additive predictive power was observed for unfavorable functional outcomes (Supplementary Table 6). The TMAO levels at days 2 and 7 did not have predictive power for 90-day major ischemic events (AUC = 0.58, *p* = 0.31; AUC = 0.57, *p* = 0.54) or unfavorable functional outcomes (AUC = 0.52, *p* = 0.68; AUC = 0.50, *p* = 0.98) but had AUCs of 0.71 (95% CI, 0.58–0.83; *p* = 0.002) and 0.71 (95% CI, 0.55–0.86; *p* = 0.013) for 12-month major ischemic events, 0.64 (95% CI, 0.51–0.76; *p* = 0.039) and 0.70 (95% CI, 0.52–0.87; *p* = 0.036) for 12-month unfavorable functional outcomes, respectively.

## Discussion

This study showed that the TMAO levels in AIS patients showed no significant changes within 24 h of AIS treatment (higher than those in controls) but decreased significantly thereafter. Elevated baseline TMAO levels but not those of afterwards were associated with increased risks of major ischemic events and unfavorable functional outcomes after adjustments for the currently established risk factors. With a moderate predictive potential, baseline TMAO levels improved the prognostic accuracy of conventional risk factors, the NIHSS scores and the NT-proBNP levels. To the best of our knowledge, this is the first in-depth study from China to explore the dynamic changes of TMAO in AIS and identify the prognostic value of TMAO for poor stroke outcomes.

Recent studies on TMAO, a microbial metabolite, have unveiled possible pathways by which it promotes atherosclerosis and cardiovascular diseases (3, 4). Nam et al. reviewed the role of TMAO in stroke, atrial fibrillation, diabetes, coronary artery disease, chronic kidney disease, and Alzheimer's disease; the mechanisms of TMAO associated atherosclerosis and thrombosis; as well as the possible therapeutic strategies targeting TMAO (25). Finally, it was prospected that additional interventional studies were urgently needed to determine the relationships between TMAO and the development and progression of certain diseases (25). So far, little is known regarding the involvement of TMAO in AIS patients. In our previous study, AIS patients within 7 days of onset had lower TMAO levels than controls (18), which was probably because the included patients had already received stroke treatment for a few days and the treatment, especially antithrombotic agents, might reduce TMAO levels. Another study found higher TMAO levels in AIS patients within 24 h of symptom onset and a positive correlation between NIHSS score and TMAO level (19). Here, we found a significant decrease in the TMAO level after stroke, and the TMAO levels at day 7 were even lower than those of the controls. Minor stroke (defined as NIHSS scores ≤5, *N* = 125, 61.3%) was in the majority in our study and no correlation was observed between TMAO and NIHSS. Interestingly, a small cohort of 34 acute myocardial infarction patients showed that TMAO levels increased between days 1 and 3, and stabilized at day 5 (7). Moreover, the chronic-phase TMAO level in ST-segment elevation acute myocardial infarction was reported to be higher than that of the acute-phase (16). This discrepancy might be explained by the unequal impact of treatment on TMAO among different types of vascular diseases. Elevated TMAO levels could be attenuated by aspirin (20). Our analyses of different antiplatelet agents showed a significant decrease in the TMAO level, and aspirin or aspirin + clopidogrel induced a sharper drop, which suggested, in line with recent studies (4, 20, 26–28) linking TMAO to platelet reactivity, that TMAO might be a novel therapeutic target. However, considering the effects of confounding factors, it remains unclear which antiplatelet agent better reduces TMAO levels and possibly contributes to a better prognosis. Additional investigations are warranted to further examine the correlations among TMAO levels, antiplatelet agents and prognosis.

Shafi et al. (29) reported that TMAO increased cardiovascular mortality in white but not in black hemodialysis patients, indicating that the effect of TMAO on outcomes might differ by race. Nevertheless, consistent with findings on cardiovascular diseases and from Western countries, we found that elevated TMAO levels at an earlier period were associated with increased risks of major ischemic events and unfavorable functional outcomes beyond the currently established risk factors, while the TMAO levels at days 2 and 7 failed to have similarly strong associations. Patients subjected to both intravenous thrombolysis and endovascular treatments tended to suffer more major ischemic events and unfavorable functional outcomes than those subjected to other therapies in our study. It might be explained by the unequal severities of stroke. Intriguingly, the chronic-phase but not the acute-phase TMAO levels in ST-segment elevation acute myocardial infarction were found to independently predict future cardiovascular events (16), opposite to our findings. The dynamic changes of TMAO might differ among different types of vascular diseases, but higher TMAO levels still portended a poor outcome. One study from Germany enrolled ischemic stroke patients within 7 days of onset and linked higher TMAO levels to an increased risk of subsequent cardiovascular events (30). A recently published research recruited patients within 24 h of stroke onset and found that relatively high TMAO levels were associated with an increased risk of poor functional outcome (31). Another study included thrombotic, lacunar and embolic stroke patients only and demonstrated that TMAO was an independent predictor of future cardiovascular events (17). In our cohort, relationships between elevated baseline TMAO levels and increased risks of both major ischemic events and unfavorable functional outcomes were identified. In addition, we conducted with TMAO levels of different time points and observed at 90 days and 12 months, as well. The AUCs of baseline TMAO levels for predicting 90-day and 12-month major ischemic events were 0.72 and 0.76, and those for 90-day and 12-month unfavorable functional outcomes were 0.65 and 0.65, which were higher than those of 0.66 and 0.63 reported in other studies (30, 31) and those with TMAO levels at days 2 and 7.

Traditional risk factors [age (32), sex (33), serum glucose level (34), and high blood pressure (35)], NIHSS score (36) and NT-proBNP level (37) were reported to be associated with poor stroke outcomes. However, the commonly used NIHSS score for predicting stroke outcome is not recommended for those with posterior circulation strokes (38). Additionally, its reliability has been challenged, as scores from different clinicians might be inconsistent and thus confound assessments (39). In our study, baseline TMAO levels improved the prognostic accuracy of conventional risk factors, the NIHSS scores and the NT-proBNP levels, further supporting the use of TMAO as a potential prognostic marker. To date, most studies have found correlations between higher TMAO levels and increased cardiovascular risk. Further explorations are needed to determine whether interventions that reduce TMAO levels improve the prognosis of certain diseases.

The underlying mechanisms linking elevated TMAO levels to poor stroke outcomes might be speculated as follows. More than 40% of ischemic stroke is caused by large-artery atherosclerosis, while TMAO accelerates atherosclerosis with augmented macrophage cholesterol accumulation and foam cell formation (3); promoted vascular inflammation (40, 41) and endothelial dysfunction (42). TMAO was also indicated to increase oxidative stress, enhance mitochondrial impairment and inhibit the mTOR signaling, all of which damaged neurological function (43). In addition, hypertension and diabetes, which are risk factors for recurrent stroke, have been found to be promoted by TMAO. Specifically, TMAO increases plasma osmotic pressure, triggering the regulation of the TMAO-AVP-AQP-2 axis and thus eliciting greater water reabsorption (44). TMAO inhibits the hepatic insulin signaling pathway and exacerbates impaired glucose tolerance (45). Moreover, TMAO has been reported to foster platelet hyperreactivity through altering stimulus-dependent calcium signaling, thus enhancing thrombotic potential *in vivo* (4). As an event of thrombosis and thromboembolism, stroke might be induced and aggravated by elevated TMAO levels. Interestingly, recent studies revealed that both TMAO production potential (46) and susceptibility to atherosclerosis (47), thrombosis (4) were transmissible, and that gut commensals containing the choline utilization C gene (necessary for TMAO production) transmitted enhanced platelet reactivity and thrombotic potential dose-dependently (27), further supporting a role for gut microbiota-derived TMAO in increasing the risk of atherogenesis and thrombosis.

The strength of this study is that we collected blood samples in a fasting state and explored for the first time the dynamic changes of TMAO in AIS, from which we found a significant decrease. With little influence by stroke course and treatment, elevated TMAO levels before or within 24 h of AIS treatment, but not those of afterwards, had strong associations with poor stoke outcomes even after strict adjustments. Some limitations exist in this study. First, this was a single-center short-term follow-up study with a small sample size, which could be subject to selection bias and might not be sufficiently generalizable. The healthy group did not have comparable histories of hypertension, dyslipidemia, or diabetes to AIS group, which might influence the comparisons. So far, little is known regarding the influence of hypotensive, lipid-lowering and hypoglycemic medicaments on TMAO levels apart from an interesting finding showed that the current guideline-based heart failure treatment (beta-blocker, ACEi/ARB, diuretic, etc.) did not affect TMAO levels (15). It must be of great necessity to compare the pre-stroke and acute stroke to explore whether stroke itself elevates TMAO. Second, the percentages of end points were quite low after the strictly implementation of guideline-recommended secondary prevention therapies. Few stroke recurrences were observed (also probably because some fatal stroke recurrences had been classified as death events) during follow-up, which prevented us from detecting associations between TMAO and stroke recurrence. Third, although elevated TMAO levels at an earlier period and a significant decrease afterwards were observed in AIS, we should note that no observational study allows concluding any cause-and-effect relationships. Hence, our results should be interpreted with caution.

In conclusion, TMAO levels decreased with time since stroke onset and elevated TMAO levels at an earlier period portended poor stroke outcomes, improving the currently established risk factors with regard to their predictive ability. Our data broaden the potential clinical utility of TMAO as an independent prognostic marker and therapeutic target. Further carefully designed long-term follow-up prospective studies with larger sample sizes are needed to validate our findings.

## Data Availability Statement

The raw data supporting the conclusions of this article will be made available by the authors, without undue reservation, to any qualified researcher.

## Ethics Statement

The studies involving human participants were reviewed and approved by the investigational review board of Nanfang Hospital, Southern Medical University. The patients/participants provided their written informed consent to participate in this study.

## Author Contributions

JY, YH, and HZ planned and supervised the study. CT and HW drafted the manuscript and reviewed the final version. CT and ZC performed the data processing and statistical analysis. HW and XZ preprocessed the blood samples before LC/MS detection. CT, JZ, QW, and GX actively recruited patients and contributed to the data acquisition and telephone interviews. XG and RX were involved in the study design and critically revised the manuscript. All authors read and approved the final manuscript.

### Conflict of Interest

The authors declare that the research was conducted in the absence of any commercial or financial relationships that could be construed as a potential conflict of interest.
