# Supplementary material for: Dynamic Changes and Prognostic Value of Gut Microbiota-Dependent Trimethylamine-N-Oxide in Acute Ischemic Stroke
Source: Front Neurol. 2020 Jan 31;11:29. doi: 10.3389/fneur.2020.00029 (PMC7005238; doi:10.3389/fneur.2020.00029)
Supplement: Supplementary file 1 [file Data_Sheet_1.doc]

**Dynamic Changes and Prognostic Value of Gut Microbiota-Dependent Trimethylamine-N-Oxide in Acute Ischemic Stroke**

***Supplementary Materials***


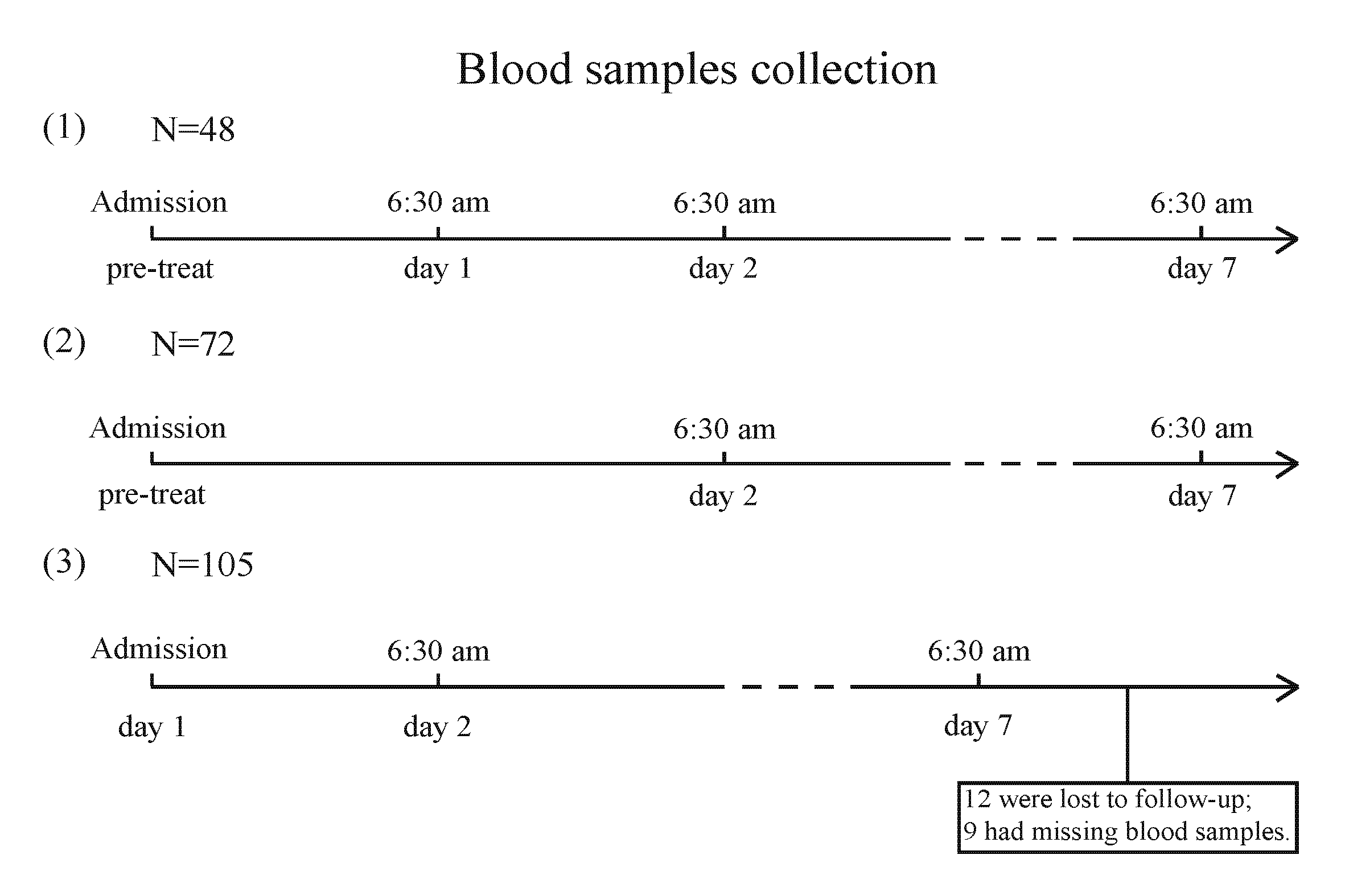


Supplementary Figure 1. Enrollment process of the study. Pre-treat indicated before AIS treatment; day 1, within 24 hours of treatment; day 2, 2 days of admission and day 7, 7 days of admission.

**
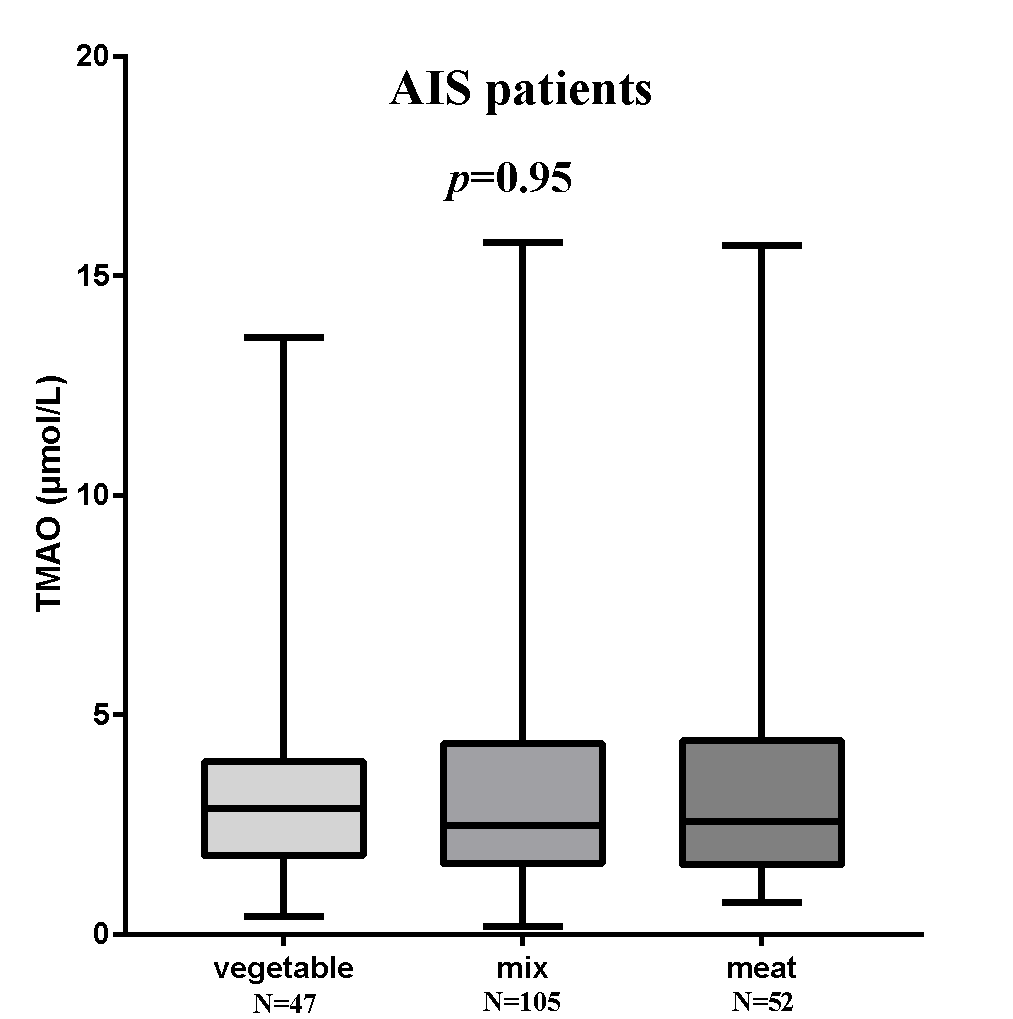
**

Supplementary Figure 2. No significant differences were found on TMAO levels among AIS patients of different dietary habits (p=0.95).


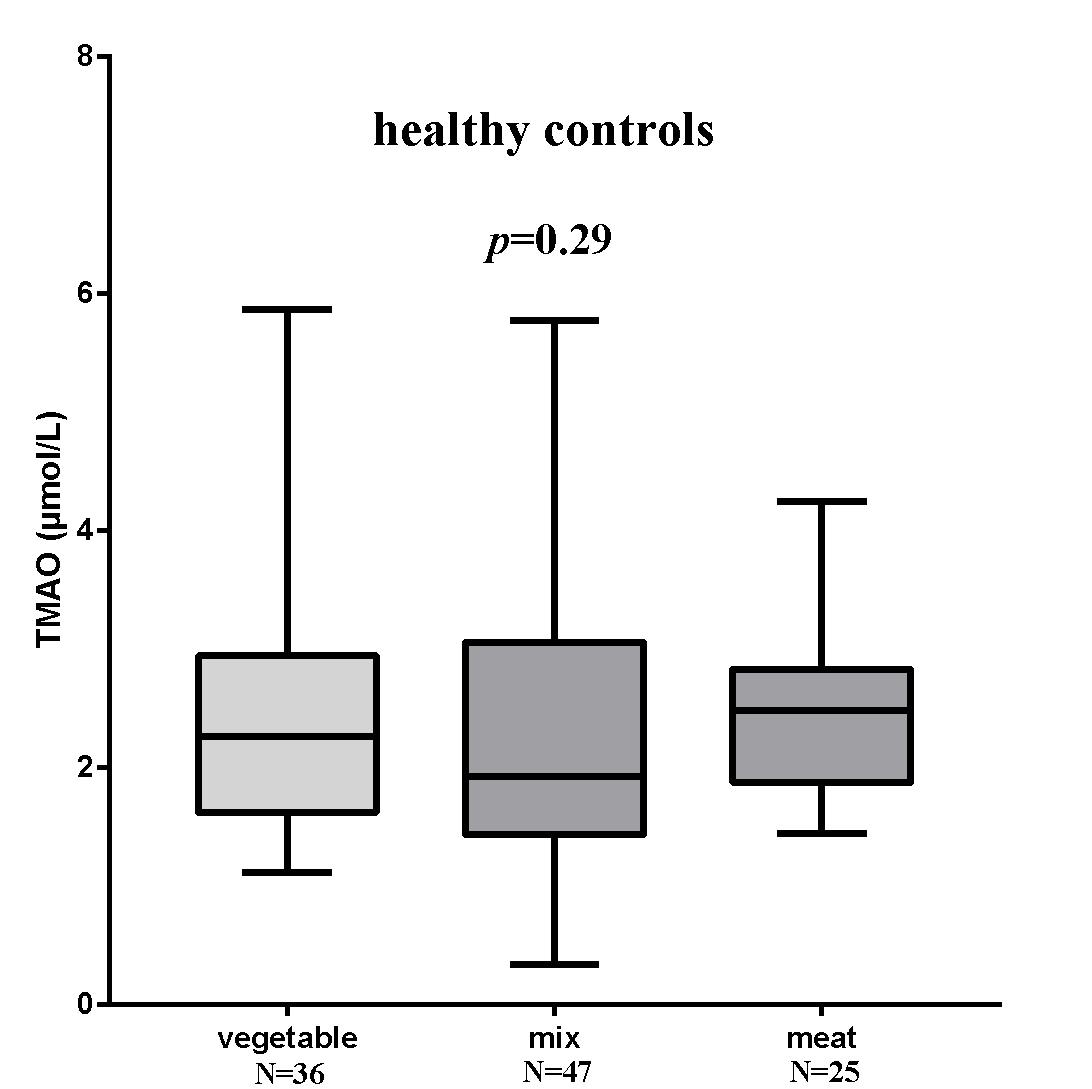


Supplementary Figure 3. No significant differences were found on TMAO levels among healthy controls of different dietary habits (p=0.29).

**
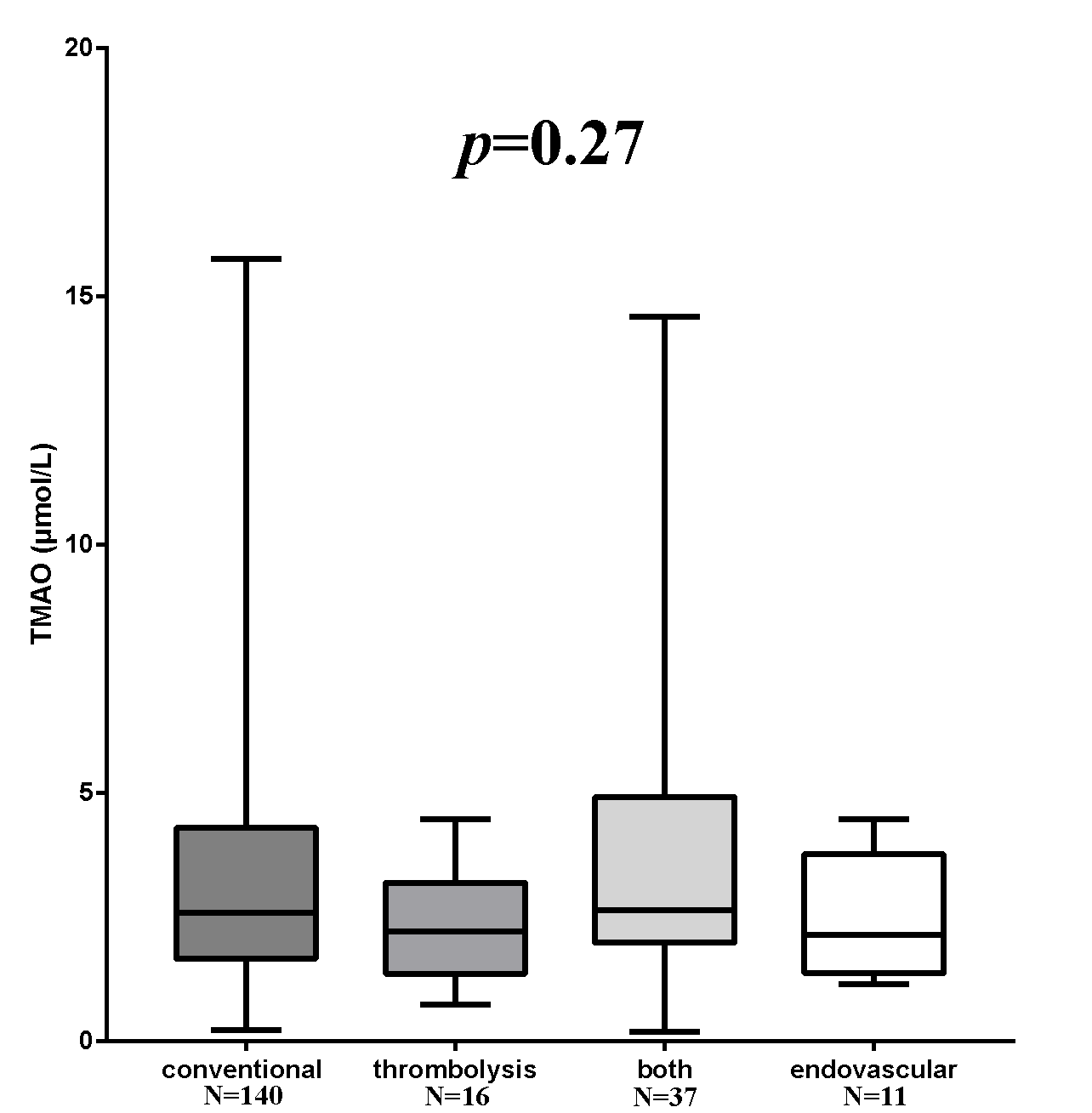
**

Supplementary Figure 4. No significant differences were found on TMAO levels among AIS patients receiving different revascularization therapies (p=0.27).

**Supplementary Table 1**. Baseline Characteristics of the Study Participants

| Characteristics | Healthy controls | AIS patients | *p* value |
| --- | --- | --- | --- |
|
| No. of subjects | 108 | 204 |  |
| Demographics |  |  |  |
| Male | 64 (59.3) | 136 (66.7) | 0.19 |
| Age, y | 59 (7) | 59 (21) | 0.76 |
| History of smoking | 37 (34.3) | 79 (38.7) | 0.44 |
| Medical history |  |  |  |
| History of hypertension | 42 (38.9) | 134 (65.7) | <0.001 |
| History of diabetes | 11 (10.2) | 59 (28.9) | <0.001 |
| History of dyslipidemia | 55 (50.9) | 76 (37.3) | 0.020 |
| Laboratory findings |  |  |  |
| WBC, ×10^9^/L | 6.35 (1.74) | 8.56 (3.62) | <0.001 |
| NEU, ×10^9^/L | 3.51 (1.06) | 5.64 (3.90) | <0.001 |
| TC, mmol/L | 5.50 (0.98) | 4.89 (1.45) | <0.001 |
| LDL, mmol/L | 3.47 (0.83) | 3.15 (1.10) | 0.001 |
| eGFR, ml/min/1.73 m^2^ | 123.96 (30.07) | 100.95 (40.64) | <0.001 |

Data are presented as number (%) or median (IQR).

**Supplementary Table 2. Baseline Characteristics of the AIS Patients With or Without** **Unfavorable Functional Outcomes at 90 Days**

| Characteristics | AIS patients | Unfavorable functional outcomes at 90 days | | *p* value |
| --- | --- | --- | --- | --- |
|  |  | Yes | No |  |
| No. of subjects | 204 | 54 | 150 |  |
| Demographics |  |  |  |  |
| Male | 136 (66.7) | 34 (63.0) | 102 (68.0) | 0.50 |
| Age, y | 59 (21) | 66.5 (24) | 57.5 (18) | <0.001 |
| History of smoking | 79 (38.7) | 15 (27.8) | 64 (42.7) | 0.054 |
| Medical history |  |  |  |  |
| History of hypertension | 134 (65.7) | 38 (70.4) | 96 (64.0) | 0.40 |
| History of diabetes | 59 (28.9) | 13 (24.1) | 46 (30.7) | 0.36 |
| History of atrial fibrillation | 27 (13.2) | 21 (38.9) | 6 (4.0) | <0.001 |
| History of coronary heart disease | 18 (8.8) | 10 (18.5) | 8 (5.3) | 0.003 |
| History of stroke | 30 (14.7) | 10 (18.5) | 20 (13.3) | 0.36 |
| History of dyslipidemia | 76 (37.3) | 22 (40.7) | 54 (36.0) | 0.54 |
| Clinical features |  |  |  |  |
| NIHSS score | 4 (9) | 14 (8) | 3 (4) | <0.001 |
| Dysphagia | 67 (32.8) | 47 (87.0) | 20 (13.3) | <0.001 |
| Revascularization therapies |  |  |  | <0.001 |
| Conventional treatments | 140 (68.6) | 21 (38.9) | 119 (79.3) |  |
| Intravenous thrombolysis | 16 (7.8) | 0 | 16 (10.7) |  |
| Intravenous thrombolysis+ endovascular treatments | 37 (18.1) | 29 (53.7) | 8 (5.3) |  |
| Endovascular treatments | 11 (5.4) | 4 (7.4) | 7 (4.7) |  |
| Stroke causes |  |  |  | <0.001 |
| Large artery atherosclerosis | 96 (47.1) | 30 (55.6) | 66 (44.0) |  |
| Small artery occlusion lacunae | 49 (24.0) | 0 | 49 (32.7) |  |
| Cardiac embolism | 32 (15.7) | 18 (33.3) | 14 (9.3) |  |
| Other cause | 7 (3.4) | 2 (3.7) | 5 (3.3) |  |
| Unknown | 20 (9.8) | 4 (7.4) | 16 (10.7) |  |
| Antiplatelet agents |  |  |  | <0.001 |
| Aspirin | 46 (22.6) | 23 (42.6) | 23 (15.3) |  |
| Clopidogrel | 49 (24.0) | 15 (27.8) | 34 (22.7) |  |
| Aspirin+clopidogrel | 109 (53.4) | 16 (29.6) | 93 (62.0) |  |
| Laboratory findings |  |  |  |  |
| NEU, ×10^9^/L | 5.64 (3.90) | 6.69 (4.71) | 5.37 (3.44) | 0.001 |
| NT-proBNP, pg/ml | 88.07 (376.49) | 233.65 (1357.01) | 68.28 (250.99) | <0.001 |
| TC, mmol/L | 4.89 (1.45) | 4.90 (1.43) | 4.88 (1.54) | 0.32 |
| eGFR, ml/min/1.73 m^2^ | 100.95 (40.64) | 98.19 (40.94) | 101.09 (40.37) | 0.48 |

Data are presented as number (%) or median (IQR).

**Supplementary Table 3. Baseline Characteristics of the AIS Patients With or Without Unfavorable Functional Outcomes at 12 Months**

| Characteristics | AIS patients | Unfavorable functional outcomes at 12 months | | *p* value |
| --- | --- | --- | --- | --- |
|  |  | Yes | No |  |
| No. of subjects | 204 | 38 | 166 |  |
| Demographics |  |  |  |  |
| Male | 136 (66.7) | 24 (63.2) | 112 (67.5) | 0.61 |
| Age, y | 59 (21) | 67 (25) | 59 (18) | 0.001 |
| History of smoking | 79 (38.7) | 14 (36.8) | 65 (39.2) | 0.79 |
| Medical history |  |  |  |  |
| History of hypertension | 134 (65.7) | 24 (63.2) | 110 (66.3) | 0.72 |
| History of diabetes | 59 (28.9) | 10 (26.3) | 49 (29.5) | 0.70 |
| History of atrial fibrillation | 27 (13.2) | 17 (44.7) | 10 (6.0) | <0.001 |
| History of coronary heart disease | 18 (8.8) | 7 (18.4) | 11 (6.6) | 0.021 |
| History of stroke | 30 (14.7) | 8 (21.1) | 22 (13.3) | 0.22 |
| History of dyslipidemia | 76 (37.3) | 13 (34.2) | 63 (38.0) | 0.67 |
| Clinical features |  |  |  |  |
| NIHSS score | 4 (9) | 15 (9) | 3 (5) | <0.001 |
| Dysphagia | 67 (32.8) | 35 (92.1) | 32 (19.3) | <0.001 |
| Revascularization therapies |  |  |  | <0.001 |
| Conventional treatments | 140 (68.6) | 14 (36.8) | 126 (75.9) |  |
| Intravenous thrombolysis | 16 (7.8) | 0 | 16 (9.6) |  |
| Intravenous thrombolysis+ endovascular treatments | 37 (18.1) | 21 (55.3) | 16 (9.6) |  |
| Endovascular treatments | 11 (5.4) | 3 (7.9) | 8 (4.8) |  |
| Stroke causes |  |  |  | <0.001 |
| Large artery atherosclerosis | 96 (47.1) | 18 (47.4) | 78 (47.0) |  |
| Small artery occlusion lacunae | 49 (24.0) | 0 | 49 (29.5) |  |
| Cardiac embolism | 32 (15.7) | 14 (36.8) | 18 (10.8) |  |
| Other cause | 7 (3.4) | 2 (5.3) | 5 (3.0) |  |
| Unknown | 20 (9.8) | 4 (10.5) | 16 (9.6) |  |
| Antiplatelet agents |  |  |  | 0.003 |
| Aspirin | 46 (22.6) | 14 (36.8) | 32 (19.3) |  |
| Clopidogrel | 49 (24.0) | 13 (34.2) | 36 (21.7) |  |
| Aspirin+clopidogrel | 109 (53.4) | 11 (28.9) | 98 (59.0) |  |
| Laboratory findings |  |  |  |  |
| NEU, ×10^9^/L | 5.64 (3.90) | 6.69 (4.87) | 5.45 (3.72) | 0.004 |
| NT-proBNP, pg/ml | 88.07 (376.49) | 279.45 (1470.51) | 71.73 (265.36) | 0.001 |
| TC, mmol/L | 4.89 (1.45) | 4.91 (1.36) | 4.88 (1.49) | 0.58 |
| eGFR, ml/min/1.73 m^2^ | 100.95 (40.64) | 102.40 (44.47) | 100.78 (40.46) | 0.90 |

Data were presented as number (%) or median (IQR).

**Supplementary Table 4.** Univariate and Multivariate Logistic Regression Analyses for Major Ischemic Events at 90 Days and at 12 Months

| Variables | Univariate analysis | Multivariate analysis | | | | | | | | | | |
| --- | --- | --- | --- | --- | --- | --- | --- | --- | --- | --- | --- | --- |
|  | *p* value | Model 1 | | Model 2 | | Model 3 | | | | Model 4 | | |
|  |  | OR  (95% CI) | *p* value | OR  (95% CI) | *p* value | OR  (95% CI) | *p* value | | OR  (95% CI) | | | *p* value |
| Major ischemic events at 90 days (N=26) | | | | | | | | | | | | |
| Baseline TMAO  (log_2_-transformed) | 0.001 | 2.21  (1.41-3.47) | 0.001 | 2.26  (1.41-3.60) | 0.001 | 2.32  (1.43-3.78) | 0.001 | 2.62  (1.55-4.45) | | | <0.001 | |
| History of atrial fibrillation | 0.001 | - | - | 5.09  (1.87-13.90) | 0.001 |  |  |  | | |  | |
| History of stroke | 0.017 | - | - |  |  | 2.96 (1.02-8.59) | 0.046 | 3.46  (1.12-10.75) | | | 0.032 | |
| NIHSS score | <0.001 | - | - | - | - | 1.11  (1.05-1.17) | <0.001 | 1.08  (1.02-1.14) | | | 0.009 | |
| NEU | 0.002 | - | - | - | - | - | - | 1.19  (1.02-1.39) | | | 0.024 | |
| NT-proBNP | 0.007 | - | - | - | - | - | - | 1.00  (1.00-1.00) | | | 0.022 | |
| Major ischemic events at 12 months (N=37) | | | | | | | | | | | | |
| Baseline TMAO  (log_2_-transformed) | <0.001 | 2.74  (1.78-4.21) | <0.001 | 2.80  (1.80-4.36) | <0.001 | 3.04  (1.91-4.84) | <0.001 | 3.59  (2.12-6.09) | | | <0.001 | |
| History of atrial fibrillation | 0.002 | - | - | 4.59  (1.74-12.08) | 0.002 |  |  | 3.79  (1.30-11.05) | | | 0.015 | |
| NIHSS score | 0.001 | - | - | - | - | 1.09  (1.04-1.15) | <0.001 |  | | |  | |
| NEU | 0.001 | - | - | - | - | - | - | 1.29  (1.11-1.49) | | | 0.001 | |

-, not included in the model.

Model 1: unadjusted;

Model 2: adjusted for age; sex; history of smoking, hypertension, diabetes, atrial fibrillation, coronary heart disease, stroke and dyslipidemia; and eGFR;

Model 3: adjusted for all factors in model 2, plus revascularization therapies, stroke etiologies, dysphagia, NIHSS score and antiplatelet agents;

Model 4: adjusted for all factors in model 3, plus NEU, NT-proBNP and TC levels.

**Supplementary Table 5.** Univariate and Multivariate Logistic Regression Analyses for Unfavorable Functional Outcomes at 90 Days and at 12 Months

| Variables | Univariate analysis | Multivariate analysis | | | | | | | | | | | |
| --- | --- | --- | --- | --- | --- | --- | --- | --- | --- | --- | --- | --- | --- |
|  | *p* value | Model 1 | | Model 2 | | Model 3 | | | | | Model 4 | | |
|  |  | OR  (95% CI) | *p* value | OR  (95% CI) | *p* value | OR  (95% CI) | *p* value | | | OR  (95% CI) | | *p* value | |
| Unfavorable functional outcomes (mRS score ≥3) at 90 days (N=54) | | | | | | | | | | | | | |
| Baseline TMAO  (log_2_-transformed) | 0.02 | 1.44  (1.06-1.97) | 0.02 | 1.43  (1.02-2.01) | 0.039 | 2.33  (1.25-4.33) | | 0.007 | 2.89  (1.46-5.71) | | | | 0.002 |
| Age | <0.001 | - | - | 1.04 (1.01-1.07) | 0.021 | 1.09 (1.03-1.14) | | 0.001 | 1.10 (1.04-1.16) | | | | <0.001 |
| History of atrial fibrillation | <0.001 | - | - | 12.71 (4.41-36.62) | <0.001 |  | |  |  | | | |  |
| Dysphagia | <0.001 | - | - | - | - | 17.27 (4.25-70.22) | | <0.001 | 16.19 (4.04-64.93) | | | | <0.001 |
| NIHSS score | <0.001 | - | - | - | - | 1.29 (1.12-1.47) | | <0.001 | 1.27 (1.12-1.43) | | | | <0.001 |
| NEU | <0.001 | - | - | - | - | - | | - | 1.24 (1.03-1.51) | | | | 0.027 |
| Unfavorable functional outcomes (mRS score ≥3) at 12 months (N=38) | | | | | | | | | | | | | |
| Baseline TMAO  (log_2_-transformed) | 0.012 | 1.58  (1.10-2.26) | 0.012 | 1.67 (1.14-2.44) | 0.009 | 2.18  (1.30-3.68) | | 0.003 | 2.58  (1.50-4.46) | | | | 0.001 |
| History of atrial fibrillation | <0.001 | - | - | 14.35 (5.51-37.35) | <0.001 | 4.70 (1.39-15.94) | | 0.013 | 10.54 (2.48-44.76) | | | | 0.001 |
| Dysphagia | <0.001 | - | - | - | - | 18.42 (4.40-77.19) | | <0.001 | 23.78 (4.99-113.29) | | | | <0.001 |
| NIHSS score | <0.001 | - | - | - | - | 1.10 (1.02-1.19) | | 0.012 | 1.10 (1.02-1.19) | | | | 0.019 |
| NEU | <0.001 | - | - | - | - | - | | - | 1.26 (1.06-1.48) | | | | 0.007 |

-, not included in the model.

Model 1: unadjusted;

Model 2: adjusted for age; sex; history of smoking, hypertension, diabetes, atrial fibrillation, coronary heart disease, stroke and dyslipidemia; and eGFR;

Model 3: adjusted for all factors in model 2, plus revascularization therapies, stroke etiologies, dysphagia, NIHSS score and antiplatelet agents;

Model 4: adjusted for all factors in model 3, plus NEU, NT-proBNP and TC levels.

**Supplementary Table 6.** Areas Under the Curves of Selected Predictors for Unfavorable Functional Outcomes at 90 Days and at 12 Months

| Predictors | Unfavorable Functional Outcomes (mRS score ≥3) at 90 Days | | | Unfavorable Functional Outcomes (mRS score ≥3) at 12 Months | | | |
| --- | --- | --- | --- | --- | --- | --- | --- |
|  | AUC | 95% CI | *p* value | | AUC | 95% CI | *p* value |
| Baseline TMAO | 0.65 | 0.54-0.71 | 0.005 | | 0.65 | 0.56-0.75 | 0.003 |
| Conventional risk factors | 0.68 | 0.60-0.77 | <0.001 | | 0.71 | 0.61-0.81 | <0.001 |
| NIHSS score | 0.89 | 0.84-0.95 | <0.001 | | 0.87 | 0.79-0.94 | <0.001 |
| NT-proBNP | 0.67 | 0.59-0.75 | <0.001 | | 0.67 | 0.57-0.76 | <0.001 |
| TMAO+Conventional | 0.75 | 0.66-0.83 | <0.001 | | 0.78 | 0.69-0.87 | <0.001 |
| TMAO+NIHSS | 0.91 | 0.85-0.97 | <0.001 | | 0.90 | 0.83-0.96 | <0.001 |
| TMAO+NT-proBNP | 0.66 | 0.58-0.74 | <0.001 | | 0.70 | 0.61-0.79 | <0.001 |
| Pre-treat TMAO (N=120) | 0.63 | 0.53-0.73 | 0.016 | | 0.64 | 0.53-0.74 | 0.025 |
